# Sequencing and Characterisation of an Extensive Atlantic Salmon (*Salmo salar* L.) MicroRNA Repertoire

**DOI:** 10.1371/journal.pone.0070136

**Published:** 2013-07-29

**Authors:** Michaël Bekaert, Natalie R. Lowe, Stephen C. Bishop, James E. Bron, John B. Taggart, Ross D. Houston

**Affiliations:** 1 Institute of Aquaculture, School of Natural Sciences, University of Stirling, Stirling, Scotland, United Kingdom; 2 The Roslin Institute and Royal (Dick) School of Veterinary Studies, University of Edinburgh, Midlothian, Scotland, United Kingdom; Aberystwyth University, United Kingdom

## Abstract

Atlantic salmon (*Salmo salar* L.), a member of the family Salmonidae, is a totemic species of ecological and cultural significance that is also economically important in terms of both sports fisheries and aquaculture. These factors have promoted the continuous development of genomic resources for this species, furthering both fundamental and applied research. MicroRNAs (miRNA) are small endogenous non-coding RNA molecules that control spatial and temporal expression of targeted genes through post-transcriptional regulation. While miRNA have been characterised in detail for many other species, this is not yet the case for Atlantic salmon. To identify miRNAs from Atlantic salmon, we constructed whole fish miRNA libraries for 18 individual juveniles (fry, four months post hatch) and characterised them by Illumina high-throughput sequencing (total of 354,505,167 paired-ended reads). We report an extensive and partly novel repertoire of miRNA sequences, comprising 888 miRNA genes (547 unique mature miRNA sequences), quantify their expression levels in basal conditions, examine their homology to miRNAs from other species and identify their predicted target genes. We also identify the location and putative copy number of the miRNA genes in the draft Atlantic salmon reference genome sequence. The Atlantic salmon miRNAs experimentally identified in this study provide a robust large-scale resource for functional genome research in salmonids. There is an opportunity to explore the evolution of salmonid miRNAs following the relatively recent whole genome duplication event in salmonid species and to investigate the role of miRNAs in the regulation of gene expression in particular their contribution to variation in economically and ecologically important traits.

## Introduction

MicroRNAs (miRNAs) are ubiquitous non-protein coding short RNA molecules (18-26 nucleotides) which play an important role in the post-transcriptional regulation of gene expression [1,2]. They act via binding to the 3’ UTR region of the target mRNAs, resulting in mRNA degradation or translation inhibition [3]. The expression of miRNA is under tight regulation in specific tissues and developmental stages in mammals [4], flies [5], worms [6] and frogs [7]. miRNAs are involved in the control of diverse processes including animal development and growth [8], signal transduction [9], disease [10,11] and virus-induced immune defence [12,13]. The genes encoding miRNA are initially transcribed by RNA polymerase II to generate primary miRNAs (pri-miRNAs). These pri-miRNAs are processed to release miRNA precursors (pre-miRNAs) approximately 70 nt in length with characteristic hairpin structures. Pre-miRNAs are then exported from the nucleus to the cytoplasm. The pre-miRNA hairpin is then cleaved to generate a double-stranded miRNA duplex with a characteristic 3’ two nucleotide overhang. Subsequently, the double-stranded miRNA duplex is separated and one strand is selected as the mature miRNA, whereas most of the other strand, termed mature star-sequence, is degraded [14].

Before the advent of high-throughput sequencing technology the number of known miRNAs was limited to approximately 100, discovered by laborious molecular cloning and Sanger sequencing methods. More recent studies have demonstrated that high-throughput sequencing can be applied to successfully discover even low abundance miRNAs in different species [15,16]. A single mature miRNA can bind to multiple different mRNA transcripts (up to 200 genes) while each mRNA can have recognition sites for more than one miRNA [17]. Over 30% of the human protein-coding genes are believed to be negatively regulated by miRNA [18,19], which highlights the significance of their role in transcriptional and post-transcriptional regulation of gene expression [2,3].

Across all species, in excess of 25,000 mature miRNAs have been reported and deposited in miRBase [20]. The most recent release (release 19, August 2012) contains 2,042 mature miRNAs from *Homo sapiens* and 368 mature miRNA from *Caenorhabditis elegans* (nematode). Fish species are under-represented, with data available from *Danio rerio* (zebrafish; 247 mature miRNAs), *Oryzias latipes* (medaka; 147 mature miRNAs), *Cyprinus* *carpio* (common carp; 146 mature miRNAs), *Tetraodon nigroviridis* (Tetraodon; 109 mature miRNAs), *Fugu rubripes* (fugu; 108 mature miRNAs), *Paralichthys* *olivaceus* (olive flounder; 38 mature miRNAs) and *Hippoglossus* *hippoglossus* (Atlantic halibut; 1 mature miRNA).

*Salmo salar* (Atlantic salmon; family Salmonidae) is an economically important species for both wild fisheries and aquaculture production. Genomic resources for the species are advanced compared to other farmed fish, but lag behind terrestrial livestock and model species. The *S. salar* genome is in the process of being sequenced and assembled [21] and a first draft of the genome sequences is available (NCBI Assembly GCA_000233375.1). The salmonid genome is derived from an ancestral whole-genome duplication event that occurred between 25 and 100 million years ago, and is considered to exhibit ‘residual tetraploidy’ [22]. Large regions of chromosome homeology both complicate genomic analyses and provide an interesting model for the study of many facets of re-diploidisation following a whole genome duplication event. One interesting aspect concerns the role that miRNAs may play in the control of duplicate gene expression and subsequent downstream effects on the phenotype. To date experimental exploration of miRNAs in salmonids has been confined to *Oncorhynchus mykiss* (rainbow trout) where 210 miRNAs were cloned and sequenced from various tissues [23] and more recently, high throughput sequencing has been used [24] to catalogue 496 miRNAs from unfertilised ova. Information for Atlantic salmon is more sparse, with just two recent *in silico* interrogations of publically available EST sequences being published, reporting computational predictions for 307 [25] and 102 [26] putative miRNAs.

The aim of the current study was to discover *S. salar* miRNAs empirically and to begin to investigate their functional roles. The primary objectives were to sequence and characterise an extensive miRNA repertoire and to profile a reference expression level in *S. salar* fry. Identifying miRNA targets is an important step in studying miRNA functions. Gene targets have conserved perfect or near-perfect complementary sites to miRNAs [2,27]. Based on sequence complementarity between miRNAs and mRNAs, computational approaches can be used as a powerful strategy to predict miRNA targets [2,28,29]. Consequently, a secondary objective of this study was the *in silico* prediction of miRNA targets from the *S. salar* transcriptome. This miRNA discovery and characterisation phase is a first step to understanding the role of miRNA in regulating gene expression and downstream biological processes in *S. salar*.

## Results

### *De-novo* identification of miRNAs

**Figure 1:**
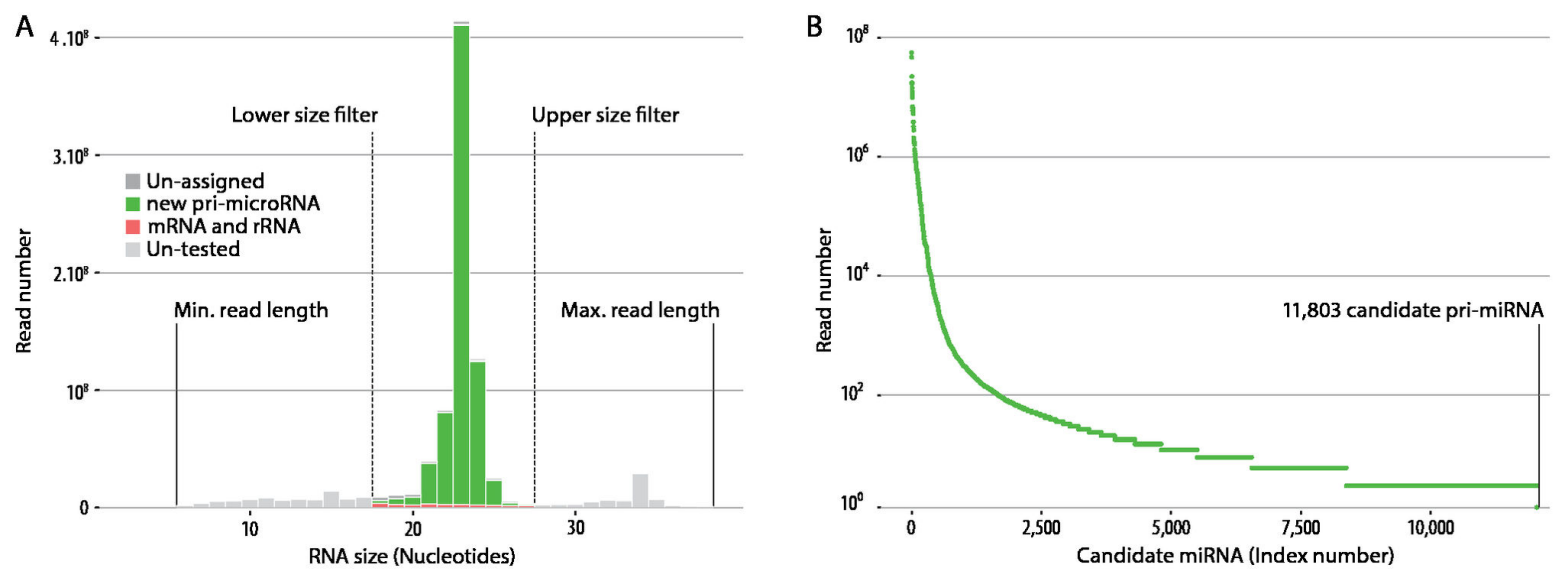
Small RNA and candidate miRNAs read number distributions. (A) Size distribution of the captured small RNA. Low quality reads or reads with ambiguous bases were discarded. Each sequence (from 5 to 37 nt long) and its count number were generated. 2,477,426 unique sequences were identified. Only the sequences between 16 and 28 nt were subsequently used. (B) Read number distribution of the 11,803 putative pri-miRNAs predicted my miRanalyzer.

Small RNA transcriptomes from 18 *S. salar* fry were analysed using high-throughput sequencing (data made available through the NCBI BioProject accession number SRP017393). In total, 354,505,167 paired-ended reads (37 nucleotides long) were obtained from the 18 homogenised whole *S. salar* fry. Low quality reads, reads with ambiguous bases, and non-identical paired-end reads were discarded (6.83%). For the remaining 330,292,464 reads each unique sequence (5-37 nt long) and its count number were generated, with 2,477,426 unique sequences being identified (Figure 1A Table S1). Sequences between 16 and 28 nt long (1,374,027 sequences, 55% of total representing 76% of the read counts) were matched against *S. salar* genome assembly (NCBI Assembly GCA_000233375.1) using miRanalyzer [30], to identify potential miRNA genes. This produced 11,803 predicted pri-miRNAs with read counts ranging between 1 and 56,087,898 (Figure 1B).

A total of 946 candidate pri-miRNAs with read counts greater than 10 reads per sample (see criteria from [20]) were filtered using RepeatMasker [31] to identify and remove repeats and low complexity sequences (Tables 1 & S1). Following this, 888 robust pri-miRNAs were retained for further analysis. Collectively these filtered sequences accounted for 72.4% of the read count (sequences between 16 and 28 nt long) and had a median read count of 5,007.

**Table 1:** Repeat elements identified using the repeat masker.

**RepeatMasker elements**	**Number of occurrences**
Retro-elements	
SINEs	2
LINEs: L2/CR1/Rex	1
LTR elements: Gypsy/DIRS1	5
DNA transposons	
hobo-Activator	2
Tc1-IS630-Pogo	2
Tourist/Harbinger	1
Small RNA (tRNA)	39
Simple repeats	6

The final dataset of putative miRNA was depleted of 58 candidate repetitive elements (details presented Table S1).

### Identification of conserved miRNAs in *S. salar*

The 888 candidate pri-miRNAs were BLASTN searched against the mature miRNA sequences in miRBase (release 19) and separately against reported *O. mykiss*-specific miRNAs [23,24]. This resulted in 459 pri-miRNAs (51.5% of *S. salar* candidates) being identified with high confidence within miRBase (e-value < 0.5; Table S2). A further 13 pri-miRNAs (1.5%) were identified as likely homologues of *O. mykiss* miRNAs (Table 2). The 416 remaining pri-miRNAs (47% of candidates) were classified as novel (Table S2). Overall, 547 mature miRNAs were generated from 766 pre-miRNA sequences covering 888 pri-miRNA genes. All miRNAs possessed a perfect two nt 3’ overhang generated by Dicer cleavage. The sequences and the secondary structures of these pri-miRNAs are shown in Table S2.

**Table 2:** Conserved mature miRNA sequences identified as having homologues in *O. mykiss* only; names, sequences, references.

**Family**	**Mature miRNA**	***O. mykiss* homolog**	**E-value**	**Sequence (5’-3’)**	**Reference**
miR-145	ssa-miR-145-5p	omy-miR-145-3p	0.3	GGATTCCAAGAAATGCT	[24]
nov-117	ssa-nov-117-3p	omy-miR-nov117-5p	0.36	TGTCTTTCACATTCTCT	[24]
	ssa-nov-117-3p	omy-miR-nov117-5p	0.36	GTCTGTGTCTATTGTCTCT	[24]
nov-14	ssa-nov-14-3p	omy-miR-nov14-5p	0.36	ACCTGTGCTCACTGTAG	[24]
nov-208	ssa-nov-208-3p	omy-miR-nov208-5p	0.35	AGTCCTGAGCTATGGCTGG	[24]
nov-70	ssa-nov-70-3p	omy-miR-nov70-3p	0.36	TCTGTTTCTCTGTGTGT	[24]
nov-71	ssa-nov-71-5p	omy-miR-nov71-3p	0.36	TCTGTTTGTGCTGTCTTGC	[24]
nov-79	ssa-nov-79-5p	omy-miR-nov79-5p	0.0000047	GACTTGGTCAAAGCTCCTCAG	[24]
miR-205	ssa-miR-205-3p	omy-miR-205	0.044	CACACTCCCGAGGACTGAAG	[23]
miR-21	ssa-miR-21a-3p	omy-miR-21-aa	0.044	AGCTCACCAGATCAGGTG	[23]

### Comparative analysis of salmonid miRNA sequences

We compared our miRNA sequences to those identified or predicted in previous studies in salmonid species and examined the overlap [23–26]. Before making any comparison, we applied RepeatMasker to identify nucleotide repetitions and other structured small RNAs (5S, tRNA, etc.). Our data are consistent with previous miRNA predictions for *S. salar* with the vast majority of predicted miRNAs being verified by the sequence data in this study (Table 3). A high degree of conservation between *S. salar* and *O. mykiss* miRNAs was demonstrated, with all the 210 cloned *O. mykiss* miRNAs [23] being retrieved in our dataset (Table 3). Only 85 out of 486 miRNAs from the *O. mykiss* unfertilised egg miRNA dataset [24] were not detected in this study (Table 3).

**Table 3:** Comparison of the miRNA with predicted and cloned mature miRNAs from previously published studies in salmonid species.

	**Published**	**Masked out**	**Hit**	**Not retrieved**	**Source**
Predicted *S. salar*	75	15	48	12	[26]
Predicted *S. salar*	307	51	248	8	[25]
Cloned *O. mykiss*	210	0	210	0	[23]
Sequenced *O. mykiss*	496	10	401	85	[24]

### miRNA abundance and genomic distribution

**Figure 2:**
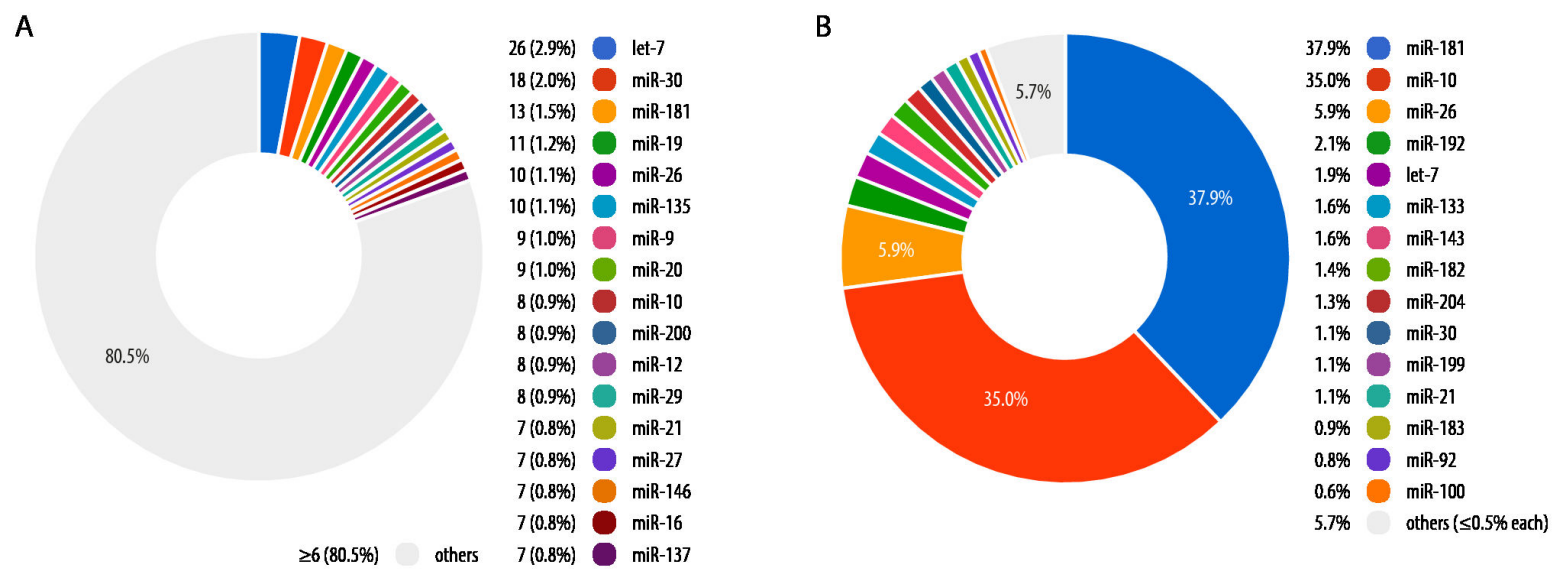
Pie charts of the novel miRNA abundance. (A) Distribution of the miRNA gene number by family. The top 17 families (over 6 genes) represents 19.5% of all the miRNA genes. All the “others” (6 genes and under) represents overall 80.5% of all the miRNA genes and less than 0.7% individually. (B) Distribution of the miRNA abundance by miRNA family. The top 15 miRNA families (relative expression over 0.5%) represents 94.3% of the expressed miRNA. All the “others” (miRNA representing individually 0.5% of the global abundance and under) represents only 5.7% of the overall expressed miRNA.

The putative gene copy number for each miRNA was estimated by alignment of candidate miRNA sequences with the *S. salar* draft reference genome. Of the 888 pri-miRNA genes, there were 122 pairs of identical duplicates, i.e., 16% of the total. The 888 pri-miRNA sequences clustered into 453 families (miRNA with similar mature miRNA, as defined by the Rfam database [32]). The miRNA families exhibited a wide range of gene copy number with let-7 (26 gene copies), miR-30 (18 gene copies), and miR-181 (13 gene copies) having the most copies in the *S. salar* draft genome assembly (Figure 2A).

Three miRNAs families predominated: miR-181 (37.9% of total miRNA transcripts), miR-10 (35.0%) and miR-26 (5.9%). Detected gene copy numbers for these miRNAs were 13, 8 and 10 respectively (compared to let-7 at 1.9% of total miRNA transcripts and miR-30 at 1.1%, Figure 2B Table S3). miRNAs with fewer than seven gene copies (n = 715) represented only 15.4% of the miRNA abundance in *S. salar*. The miRNA library was not normalised, thus providing the opportunity to compare gene copy number with transcript frequency. To assess the correlation between putative gene copy number and miRNA family abundance (Table S3), a Spearman’s rank correlation coefficient was calculated between the two variables. There was a positive correlation between gene copy number and miRNA family abundance (Spearman’s Rho = 0.64, *P* < 0.001) indicating that miRNA families with multiple gene copies tend to have higher overall expression levels. The distributions of the miRNA family expression among the 18 samples were compared using DESeq [33]; no significant variation of expression was detected among either *S. salar* families or individuals.

### Predicted target genes of miRNAs

The miRNA target genes were predicted using TargetSpy [34] and RNAHybrid [35] using all 119,912 *S. salar* mRNA sequences available from the Centre for Biomedical Research (University of Victoria) website [36]. In total, 57,907 putative target genes (474,947 putative target sites) were identified by TargetSpy and 62,371 putative target genes (519,308 putative target sites) were identified by RNAhybrid. Overall, 8,065 common putative target genes (9,580 putative target sites; Table S4) were identified as possible targets for miRNA regulation (from 548 mature miRNAs). However, confirmation of the true relationships between these miRNAs and their putative targets requires functional analysis.

## Discussion

In the current study, a detailed characterisation of the miRNA profile of *S. salar* fry was performed and their potential target genes assessed. Unlike most miRNA sequencing studies where single pass reads have been generated, paired-end sequencing was used to improve read accuracy, aiding reliable discrimination between miRNAs differing by only one or two nucleotides. Guidelines for miRNA discrimination derived from RNA deep-sequencing data were established in 2011 [20]. All 888 pri-miRNAs identified in this study can be classed as high-confidence miRNAs according to their criteria.

From a comparison with miRBase it would appear that only 51.5% of the identified mature miRNAs have been previously reported. This is likely to be a reflection of the comparative under-representation of fishes in the current database and stage / state specific expression rather than an indication that the remaining sequences are necessarily unique to *S. salar*. Lack of detectable differential miRNA expression among individuals and families, reared in different tanks, suggests either that the detected miRNA profile is constitutively expressed in a conserved manner or that responses are relatively uniform between individuals in this study.

While 453 miRNA families were identified in the current experiment, just three of these accounted for approximately 78.8% of the miRNA abundance; namely miR-181 (37.9%), miR-10 (35.0%) and miR-26 (5.9%). This likely reflects the nature of the biological samples, sequences being derived from whole fry homogenates leading to overrepresentation of miRNAs from the more abundant tissues. Given the young age of the salmon fry in this experiment (approximately 4 months post-hatching), the fry are fast growing with the predominant tissue being skeletal muscle. In *O. mykiss* eggs, Ma et al. [24] identified let-7, miR-21 and miR-24 as the most abundant miRNA with 24.06%, 18.71% and 6.59% respectively. Whereas in *O. mykiss* pooled tissues [23], miR-21, miR-125 and miR 204 were the most abundant miRNA with 35%, 14% and 10% respectively. In the current study, let-7 and miR-21 are also among the most highly expressed miRNAs with 1.9% and 1.1% of the relative abundance respectively.

All of the three most abundant *S. salar* miRNAs have been found to be involved in regulation of developmental processes, these observations being conserved across several species. miR-181 regulates haematopoiesis development and plays a role in T-lymphocyte maturation and the sensitivity of lymphocytes to T-cell receptor stimulation [37]. miR-10 regulates the highly-conserved transcription factor *Hox* genes which have highly conserved roles in early development [38]. In most vertebrates studied to date, the miR-10 genes co-locate with the *Hox* cluster and, in *D. rerio*, loss of function of miR-10 points to a role in anterior–posterior patterning [39]. Finally, miR-26 contributes to myogenesis [40] and neurogenesis [41]. miR-26 expression has been suggested to be involved in *O. mykiss* embryonic development and Ramachandra et al. [42] suggested a possible link between the miR-26 response to hypoxia and the short supply of dissolved oxygen in the aquatic environment where salmonid eggs develop [42].

The *S. salar* miRNAs identified in this study may have applications for both fundamental and applied research. For example, they can be assessed as potential markers for specific functional and diagnostic applications in *S. salar* aquaculture production. Polymorphism in the miRNA genes or their target sites may underlie phenotypic variation in quantitative traits (e.g., fillet characteristics or disease resistance) and could be assessed for use in selective breeding programs to improve the efficiency of salmon production Where the precise function of a miRNA is known, artificial miRNAs may be used to suppress gene expression for the knockdown of targeted genes [2] as a potentially useful tool in salmonid research. Therefore, these miRNAs provide a platform for the elucidation of gene function via a series of hypothesis-driven studies in Atlantic salmon for aquaculture and biomedical research. This functional approach has already proven productive in the study of the role of hepatic miRNAs in insulin regulation pathways in *O. mykiss* [43].

## Conclusion

We have discovered an extensive repertoire of putative miRNA in Atlantic salmon, quantified their expression levels in basal conditions in fry and identified the location and putative copy number of the miRNA genes in the draft Atlantic salmon reference genome sequence. These *S. salar* miRNAs provide a novel resource to advance functional genome research in salmonid species.

## Materials and Methods

### Animals

Atlantic salmon fry from three families of farmed origin were reared in separate 15 L family-specific holding tanks, all supplied with de-chlorinated freshwater maintained at 10-12°C, at the UK Government’s Centre for Environment, Fisheries and Aquaculture Science (Cefas) in Weymouth. Negligible mortalities occurred during this period. At 120 days post-hatch the fry (six per family, mean weight ~0.7 g) were sampled by snap freezing in liquid nitrogen, and stored at -80°C until processed. Fish were euthanised using a non-schedule 1 method under a procedure specifically listed on the appropriate Home Office (UK) license and under approval of Cefas ethical review committee. All working procedures complied with the Animals Scientific Procedures Act [44].

### Small RNA library construction and sequencing

#### (i): Sample processing and RNA extraction

Each frozen whole fry was cut into four pieces and immediately homogenised in 7 mL TRI Reagent (Sigma-Aldrich Co., USA) using a large Polytron mechanical homogeniser (Kinematica, Switzerland). Following incubation for 5 min at room temperature, 1 mL of the homogenate was retained for RNA extraction, following manufacturer’s protocol. The RNA was precipitated with 0.5 volume RNA precipitation solution (1.2 mol/L sodium chloride; 0.8 mol/L sodium citrate sesquihydrate) and 0.5 volume isopropanol and re-suspended in nuclease free water. The RNA was quantified by spectrophotometry using the Nanodrop 1000 (Thermo, Fisher Scientific, USA), and integrity confirmed by analysis on a Bioanalyzer 2100 (Agilent Technologies, Inc., USA), all RNA integrity numbers being over 9.9 indicating high quality RNA for each sample.

#### (ii): Library preparation

Library preparation was performed according to the Illumina Truseq Small RNA preparation guide (Illumina, Inc., USA), using total RNA as start material. In brief, 5’ and 3’ RNA adapters, designed such that they would preferentially ligate small RNAs, were added to 1 µg total RNA. The adapter-ligated small RNA was then reverse transcribed and amplified through PCR. The resulting product was size selected using PAGE, where molecules sized 145-160 bp were excised from polyacrylamide gel and subsequently purified and concentrated through ethanol precipitation. The libraries were validated using a Bioanalyzer 2100 (Agilent Technologies, Inc., USA). One multiplexed barcoded pool containing all libraries was sequenced (two lanes Illumina HiSeq 2000, 37 base paired-end run) at ARK-Genomics (Roslin, UK). Raw sequence data were made available through the NCBI BioProject accession number SRP017393.

### Identification of novel miRNA candidates

All reads that were mapped to known small RNAs were removed, including: RNA from RFam 10.1 [32]; tRNA from the GtRNAdb [45]; piRNA from RNAdb [46] and mRNAs from the Reference Sequence (RefSeq) database [47]. We used an accurate machine learning algorithm to predict miRNA implemented in miRanalyzer version 0.3 [beta] [30]. Sequences between 16 and 28 nucleotides in length were aligned with the *S. salar* genome (NCBI Assembly GCA_000233375.1).

Guidelines for miRNA annotation using RNA deep-sequencing experiments were established in 2011 [20]; They require that 1) multiple reads be identified to support the presence of the mature ~22 nt miRNAs; 2) the reads map to an extended sequence region, and the sequence flanking the putative mature miRNA folds to form a miRNA precursor-like hairpin with strong pairing between the mature miRNA and the opposite arm; 3) mapped reads should not overlap other annotated transcripts; 4) reads mapping to a locus support consistent processing of the 5’-end of the mature sequence; 5) reads support the presence of mature sequences from both arms of the predicted hairpin (mature and mature star-sequences), and the putative mature sequences should base-pair with the correct 3’-overhang.

The pre-miRNA is defined as the sequence that starts at the first “bulge” (regions in which one strand of a miRNA has “extra” inserted bases with no counterparts in the opposite strand) before the 5’ mature miRNA, and ends at the corresponding position at the 3’ terminus. The minimum length of pre-miRNA is 65 nt if the flanking side of the pre-miRNA does not extend to the next bulge [30]. The secondary structures of the miRNAs were determined by the RNAfold version 2.0.7 [48] using a minimum free energy (MFE) algorithm [49].

All novel pre-miRNAs were identified based on the presence of a classic hairpin structure, Dicer cleavage pattern (a characteristic 2 nt 3’ overhang), the mature and mature star-sequences, and conservative 5’ sequence, as well as detectable expression: all candidate miRNAs with fewer than 10 read count per samples were removed. The remaining candidate miRNAs were filtered with RepeatMasker open-4.0.0 [RMLib release 20120418 and Dfam release 1.1; 31] and then aligned to a set of all known miRNAs from miRBase version 19 [20] using BLASTN [50]. These mapped reads were retained and considered as belonging to putatively homologous miRNAs (detected in other species). In the case of *S. salar*-specific miRNAs, the length of the most highly expressed read was considered as the length of the mature miRNAs [51].

### Target predictions for *S. salar* miRNAs

The 119,912 full-length transcript sequences were downloaded from the Centre for Biomedical Research (University of Victoria) website [36]. TargetSpy v 1.0 [34] and RNAHybrid v 2.1 [35] were used to predict the target genes of the miRNAs on the 3’ UTR regions of the transcripts. The TargetSpy principle of predicting miRNA target genes is based on machine learning and selected features, such as compositional, structural, and base-pairing features. The RNAHybrid algorithm, on the other hand, is based upon the identification of thermodynamically stable matches. *S. salar* xi (position) and theta (shape) parameters were 2.327203 and 0.216961 respectively, based on *S. salar* dinucleotide frequency of the 119,912 full-length transcript sequences. The predictions were filtered to make sure that the binding site presented a seed of at least 7 nt and started at the first or second position of the miRNA.

### Data deposition

The sequencing data generated in this study have been submitted to the NCBI Sequence Read Archive (SRA) and are accessible under the NCBI BioProject accession number SRP017393.

## Supporting Information

Table S1 58 miRNA precursors filtered out by RepeatMasker. Names, mature and pri-miRNA sequences, predicted secondary structure (using ViennaRNA dot-bracket notation [48]), genomic location and type of repeat or small RNA.
(CSV)

Table S2 888 miRNA precursors identified from *S. salar* genome. Names, mature and pri-miRNA sequences, predicted secondary structure (using ViennaRNA dot-bracket notation [48]) and genomic location.
(CSV)

Table S3 The 453 *S. salar* miRNA families: gene copy number and relative abundance. (CSV)

Table S4 The putative target genes of the *S. salar* miRNA families: TargetSpy and RNAhybrid detailed results and structure of the target site / microRNA binding site secondary structure (**using ViennaRNA dot-bracket notation [48]**). (CSV)
